# Phytochemicals, Antioxidant and Antimicrobial Potentials and LC-MS Analysis of *Centaurea parviflora* Desf. Extracts

**DOI:** 10.3390/molecules28052263

**Published:** 2023-02-28

**Authors:** Fatima Zohra Hechaichi, Hamdi Bendif, Chawki Bensouici, Sulaiman A. Alsalamah, Boutheina Zaidi, Mustapha Mounir Bouhenna, Nabila Souilah, Mohammed I. Alghonaim, Abderrahim Benslama, Samir Medjekal, Ashraf A. Qurtam, Mohamed Djamel Miara, Fehmi Boufahja

**Affiliations:** 1 Biodiversity and Biotechnological Techniques for Plant Resources Valorization Laboratory, Department of Natural and Life Sciences, Faculty of Sciences, University of Msila, Msila 28000, Algeria; 2 Laboratory of Ethnobotany and Natural Substances, Department of Natural Sciences, Ecole Normale Supérieure (ENS), Alger 16308, Algeria; 3 Biotechnology Research Center, UV 03, BP E7, Ali Mendjeli, Constantine 25000, Algeria; 4 Biology Department, College of Science, Imam Mohammad Ibn Saud Islamic University (IMSIU), Riyadh 11623, Saudi Arabia; 5 Faculty of Nature and Life Sciences, University of Constantine, Constantine 25000, Algeria; 6 Scientific and Technical Research Center in Physico-Chemical Analysis, Algiers 16000, Algeria; 7 Laboratory for the Optimization of Agricultural Production in Sub-Humid Zones (LOPAZS), Department of Agricultural Sciences, Faculty of Sciences, University of Skikda, Skikda 21000, Algeria; 8 Laboratory for Valorization of Natural Resources, Bioactive Molecules and Physico-Chemical and Biological Analysis, Department of Chemistry, University of Constantine, Constantine 25000, Algeria; 9 Department of Microbiology and Biochemistry, University of Msila, Msila 28000, Algeria; 10 Department and Faculty of Nature and Life Sciences, University of Tiaret, Tiaret 14000, Algeria

**Keywords:** antioxidant activity, antimicrobial, TLC, *Centaurea parviflora*, LC-MS, flavonoids, polyphenols

## Abstract

*Centaurea parviflora* (*C. parviflora*), belonging to the family Asteraceae, is an Algerian medicinal plant used in folk medicine to treat different diseases related to hyperglycemic and inflammatory disorders, as well as in food. The present study aimed to assess the total phenolic content, in vitro antioxidant and antimicrobial activity and phytochemical profile of the extracts of *C. parviflora*. The extraction of phenolic compounds from aerial parts was conducted using solvents of increasing polarity starting from methanol, resulting in crude extract (CE), to chloroform extract (CHE), ethyl acetate extract (EAE) and butanol extract (BUE). The total phenolic, flavonoid and flavonol contents of the extracts were determined using the Folin–Ciocalteu and AlCl_3_ methods, respectively. The antioxidant activity was measured with seven methods: 2,2-diphenyl-1-picrylhydrazyl (DPPH) assay, galvinoxyl free-radical-scavenging test, 2,2′-Azino-Bis(3-Ethylbenzothiazoline-6-Sulfonic Acid) (ABTS) assay, cupric reducing antioxidant capacity (CUPRAC), reducing power, Fe^+2^-phenanthroline reduction assay and superoxide-scavenging test. The disc-diffusion method aimed at testing the sensitivity of bacterial strains toward our extracts. A qualitative analysis with thin-layer chromatography of the methanolic extract was performed. Moreover, HPLC-DAD-MS was used to establish the phytochemical profile of the BUE. The BUE was found to contain high amounts of total phenolics (175.27 ± 2.79 µg GAE/mg E), flavonoids (59.89 ± 0.91 µg QE/mg E) and flavonols (47.30 ± 0.51 µg RE/mg E). Using TLC, different components such as flavonoids and polyphenols were noted. The highest radical-scavenging ability was recorded for the BUE against DPPH (IC_50_ = 59.38 ± 0.72 µg/mL), galvinoxyl (IC_50_ = 36.25 ± 0.42 µg/mL), ABTS (IC_50_ = 49.52 ± 1.54 µg/mL) and superoxide (IC_50_ = 13.61 ± 0.38 µg/mL). The BUE had the best reducing power according to the CUPRAC (A_0.5_ = 71.80 ± 1.22 μg/mL), phenanthroline test (A_0.5_ = 20.29 ± 1.16 μg/mL) and FRAP (A_0.5_ = 119.17 ± 0.29 μg/mL). The LC-MS analysis of BUE allowed us to identify eight compounds including six phenolic acids and two flavonoids: quinic acid, five chlorogenic acid derivatives, rutin and quercetin 3-*o*-glucoside. This preliminary investigation revealed that the extracts of *C. parviflora* have a good biopharmaceutical activity. The BUE possesses an interesting potential for pharmaceutical/nutraceutical applications.

## 1. Introduction

Algeria, because of its particular geographical location and climatic diversity, presents a rich and very large vegetation. There are more than 3000 plant species, 15% of which are endemic, belonging to several botanical families [1]. Several plants of the Asteraceae family are grown for their nutritional value (sunflower, artichoke, lettuce, chicory, chamomile, etc.) or as decorative plants (dahlias, asters, etc.) [2]. Indeed, it has been reported that the flowers and leaves of this family possess antibacterial, antifungal, antiviral, anti-inflammatory, antiproliferative and anti-leishmaniasis activity [3,4]. The secondary metabolite diversity of the Asteraceae explains their multiple pharmacological activities, and as a result, many species of this family are used in traditional medicine [5]. The genus *Centaurea* comprises more than 500 species, 45 of which grow naturally in Algeria, seven of which occur in the Sahara Desert [6]. *Centaurea* species are latex-free-resin or -essence plants that grow in tufts or in seedlings, usually in spring, and occur in different habitat types [7].

The main secondary metabolites of *Centaurea* species are represented by triterpenes, flavonoids and lignans, and they are also known to produce sesquiterpene lactones [8,9,10,11]. These secondary metabolites can be isolated from the leaves, aerial parts and sometimes roots of *Centaurea* [12]. 

*Centaurea parviflora* Desf. is a suffrutescent plant at its base, with a height of 40–60 cm, growing in dense, intricate bushes. The appendix of the bracts lacks a whitish scarious part or has a faintly marked scarious part with 8–12 lateral laciniures.

The highest leaves are not decurrent on the stem. The plant is characterized among the other knapweeds by its small (5 mm wide, 15 mm long), solitary flower head. The appendices are characterized by a strongly covered median spine. The flowers are purple with black, bellied, pubescent achenes with four marked streaks. *C. parviflora* is endemic to Algeria and Tunisia [13]. In Algeria, the plant is quite rare, but can be found in different biogeographical sectors from the west to the east of the country and from the coast to the highlands.

Many species of the genus *Centaurea* have been used in traditional medicine to treat various ailments and diseases. These species of the genus are used as anticancer, anti-inflammatory, antinociceptive, antipyretic, anti-arteropic, antineoplastic, anti-ulcerogenic and antimicrobial treatments. They are also used for rheumatic pain, cardiovascular problems, headaches, gastrointestinal symptoms and parasites and as a fever reliever, stimulant remedy and wound healer [6,14,15,16,17,18]. They are highly active in living systems and therefore have a strong pharmacological interest, which explains the long-term use of these plants in traditional medicine [19].

Due to their interesting health properties, polyphenols have received a growing interest and popularity, even though their amounts in most natural sources are often not sufficient for an optimal dietary intake. Phenolic compounds are commonly found in plants, and many of these effective components have several biological activities including antioxidative, anti-diabetic, anti-carcinogenic, antimicrobial, anti-allergic, anti-mutagenic and anti-inflammatory properties [20].

The present study aimed to carry out phytochemical profiling through a qualitative analysis with thin-layer chromatography and HPLC-DAD-MS of the butanolic extract and the TPCs. It also evaluated the in vitro antioxidant and antimicrobial activities of *C. parviflora*.

## 2. Results and Discussion

### 2.1. Extraction Yield

Extraction is the main step for recovering and isolating phytochemicals from plant materials. The extraction yield can be affected by the chemical nature of the phytochemicals, the method used, the solvent used, as well as the presence of interfering substances [21,22,23].

### 2.2. Qualitative Analysis

#### 2.2.1. Thin-Layer Chromatography Analysis (TLC)

After the development of the chromatogram, and when the solvent reached the upper line, the plate was removed, dried and examined under the UV lamp in order to identify the constituents present [24]. TLC profiling of the plant extracts in different solvent systems confirms the presence of a diverse group of phytochemicals.

**Figure 1:**
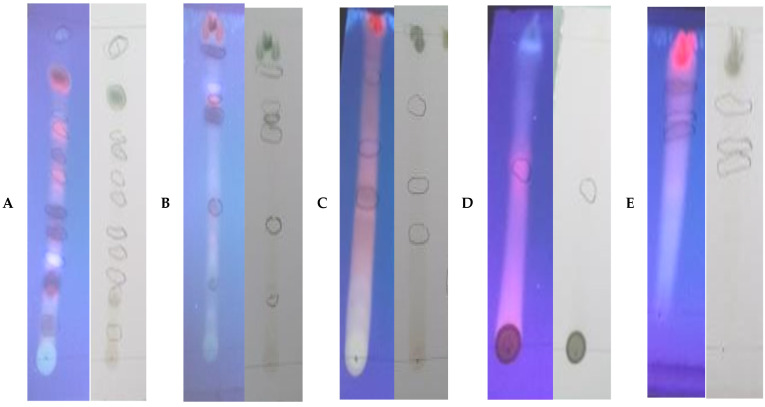
Photos of TLC results of methanolic extract of *C. parviflora.* (**A**) Toluene/Chloroform/Methanol; (**B**) Chloroform/Methanol; (**C**) Ethyl Acetate/Methanol/Water; (**D**) Methanol/Water; (**E**) Butanol/Acetic acid/Water.

The qualitative analysis made it possible to highlight numerous spots, especially those colored in blue, mauve, yellow and pink. These three stains confirm the presence of phenolic compounds [25]. According to the results (Table 1 and Figure 1), the following remarks can be noted:

The number of spots in system 1 and system 3 is higher than that in the other three systems (2, 4 and 5); this means that the plant *C. parviflora* is rich in phenolic compounds. Finally, we can conclude that the TLC results confirm the presence of phenolic compounds and flavonoids in the extracts of the *C. parviflora* plant and also reinforce what we obtained for the biological activity.

The number of spots indicates that the quantity of phenolic compounds in *C. parviflora* is higher. This confirms that this plant is rich in phenolic compounds, and these results are confirmed by our finding above (Table 1), where the plant appeared to be rich in TPC (Tukey’s HSD test: *p*-values < 0.01). Moreover, the LC-MS analysis of the butanolic fraction of *C. parviflora* mentioned six phenolic acids and 2 flavonoids, quinic acid, five derivatives of chlorogenic acid, rutin and quercetin 3-*o-*glucoside, which confirm the richness of this plant in phenolic compounds.

#### 2.2.2. LC-MS Analyses

**Figure 2:**
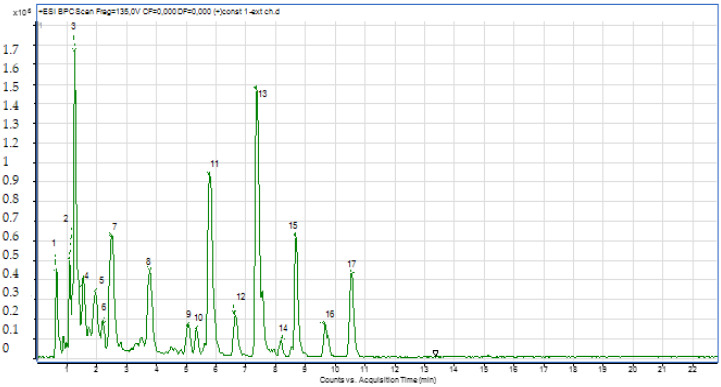
Chromatogram of the positive ionization of the butanol extract of *C. parviflora*.

**Figure 3:**
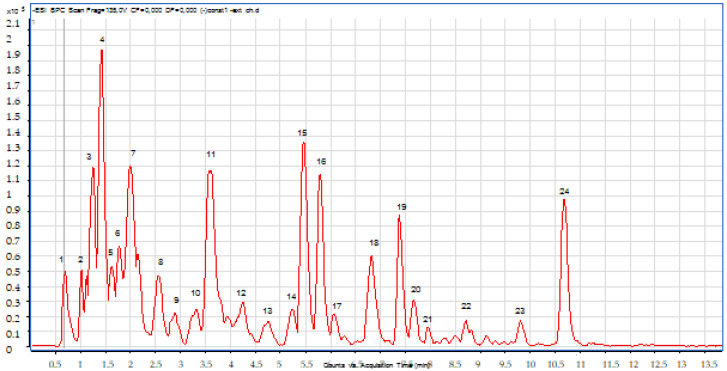
Chromatogram of the negative ionization of the butanol extract of *C. parviflora*.

A solution of 1000 ppm was prepared in methanol and injected into the LC-ESI-MS instrument. Both positive and negative ionization chromatograms show a rich concentration of the majority compounds between 0.5 and 11 min (Figure 2 and Figure 3).

**Figure 4:**
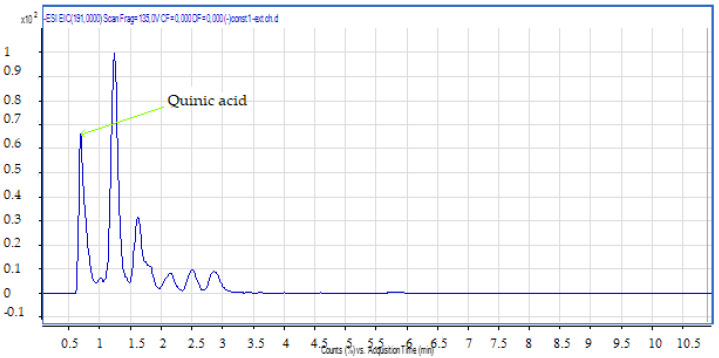
Chromatogram of quinic acid (compound **1**).

**Figure 5:**
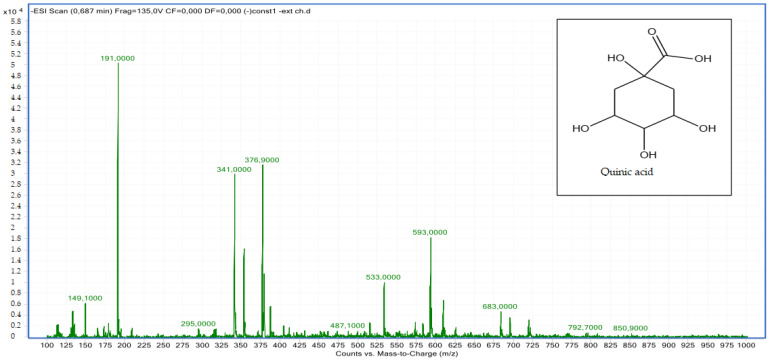
Mass spectrum of quinic acid (compound **1**).

Compound **1**: The peak that appears at a retention time of 0.68 mn with *m*/*z* = 191.00 and a crude formula of C_7_H_12_O_6_ was identified as quinic acid (Figure 4 and Figure 5).

**Figure 6:**
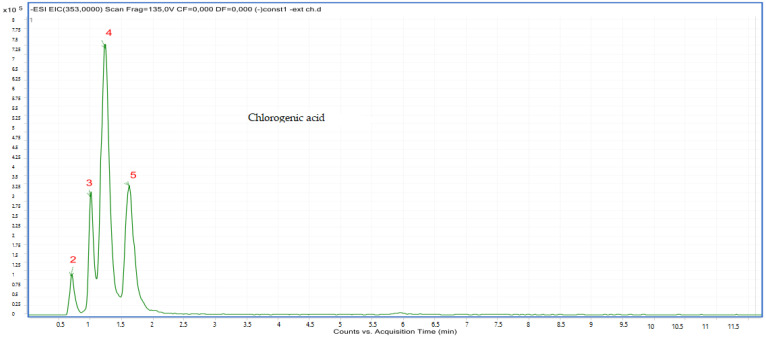
Chromatogram of chlorogenic acid (compounds **2**, **3**, **4** and **5**).

**Figure 7:**
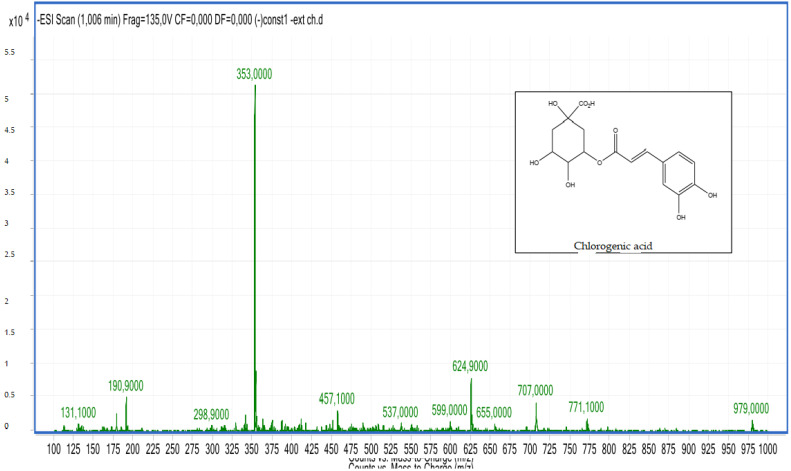
Mass spectrum of chlorogenic acid (compounds **2**, **3**, **4** and **5**).

Compounds **2**, **3**, **4** and **5**: The peak that appears at the retention times of 0.72, 1.00, 1.22, 1.62 mn with *m*/*z* = 353.00 and a crude formula of C_16_H_18_O_9_ was identified as chlorogenic acid (Figure 6 and Figure 7).

**Figure 8:**
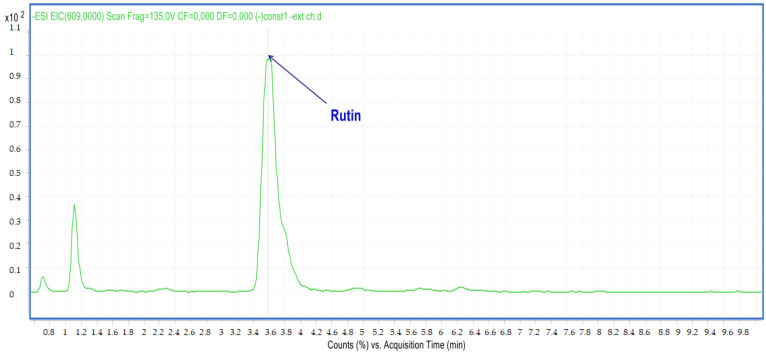
Chromatogram of rutin (compound **6**).

**Figure 9:**
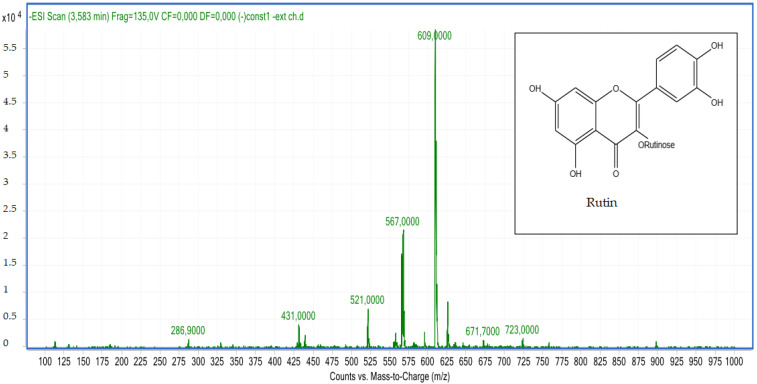
Mass spectrum of rutin (compound **6**).

Compound **6**: The peak that appears at a retention time of 3.58 mn with *m*/*z* =609.00 and a crude formula of C_27_H_30_O_16_ was identified as rutin (Figure 8 and Figure 9).

**Figure 10:**
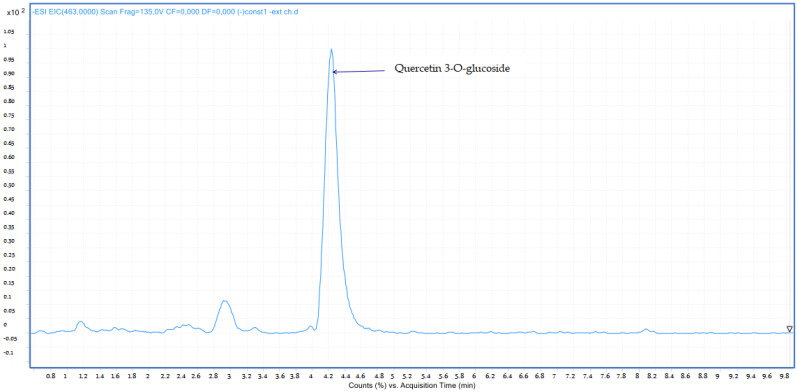
Chromatogram of quercetin 3-*O*-glucoside (compound **7**).

**Figure 11:**
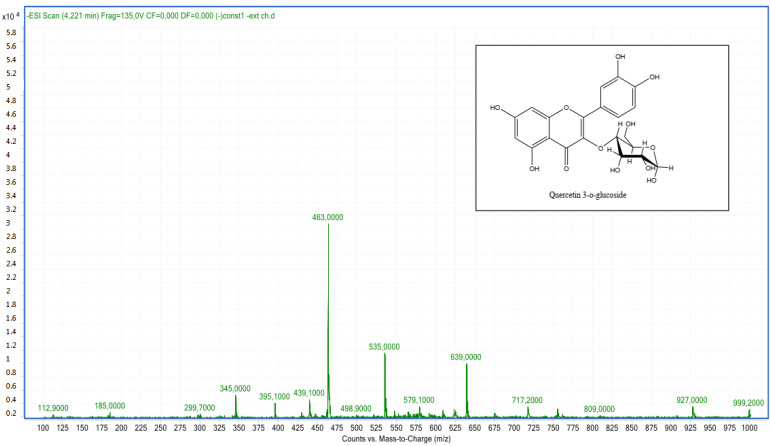
Mass spectrum of quercetin 3-*O*-glucoside (compound **7**).

Compound **7**: The peak that appears at a retention time of 4.26 mn with *m*/*z* = 463.00 and a crude formula of C_21_H_20_O_12_ has been identified as quercetin 3-*O*-glucoside (Figure 10 and Figure 11).

**Figure 12:**
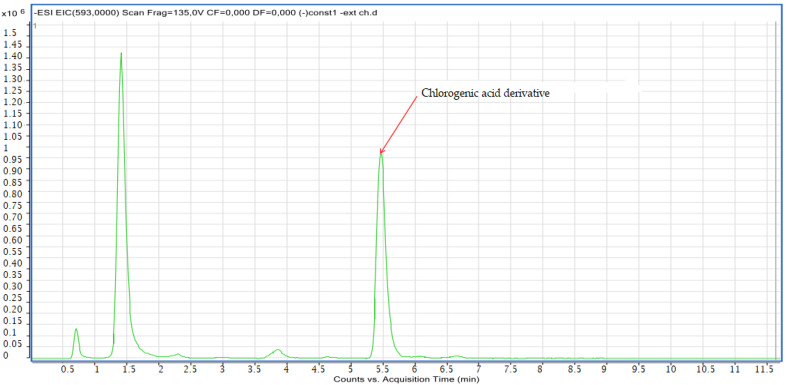
Chromatogram of chlorogenic acid derivative (compound **8**).

**Figure 13:**
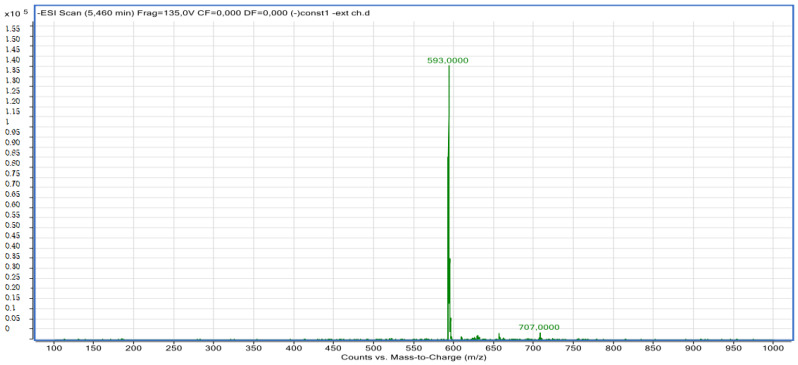
Mass spectrum of chlorogenic acid derivative (compound **8**).

Compound **8**: The peak that appears at a retention time of 5.46 mn with *m*/*z* =463.00 and a crude formula of C_30_H_10_O_14_ was identified as chlorogenic acid derivative (Figure 12 and Figure 13).

Analysis of the results from the Agilent Mass Hunter Workstation Qualitative Analysis Software B.06.00 for negative ionization in comparison with the data in the literature allowed us to identify eight compounds (Table 2).

An attempt to identify the phenolic compounds contained in the BUE was based on accurate mass comparisons [M-H] of pseudo-molecular ions with those found in accordance with the literature. The LC-MS analysis of the butanolic extract of *C. parviflora* identified eight compounds including six phenolic acids and two flavonoids: quinic acid, five derivatives of chlorogenic acid, rutin and quercetin 3-*o-*glucoside.

### 2.3. Antioxidant Activity

The total phenolic content (TPC), total flavonoid content (TFC) and total flavonol (TFOL) content and the antioxidant activity of the different extracts and fractions of *C. parviflora* were determined using different methods, and the results are shown in Table 1 and Table 3.

The difference in the polyphenol and flavonoid contents of the crude extracts and their fractions results from the difference in polarity of the organic solvents, the extraction time and temperature, the solid–liquid extraction ratio as well as the chemical and physical characteristics of the samples [30]. By comparing the results obtained, the TPC, TFC and TFOL of the different extracts were analyzed and presented in Table 1.

The results of the determination of the total phenolic compounds were obtained by extrapolating the absorbance of the extracts on the gallic acid calibration curve. The results show that the BUE is the extract richest in polyphenols with a content of 175.27 ± 2.79 µg GAE/mg of extract followed by the EAE (136.94 ± 2.94 µg GAE/mg of extract, Tukey’s HSD test: *p*-value < 0.01). In fact, the CE and CHE have the lowest phenolic contents, with 113.51 ± 2.95 and 105.47 ± 1.35 GAE/mg extract, respectively. According to the results in Table 1, the BUE is the richest in flavonoids with 59.89 ± 0.91, followed by the CE with a content of 24.49 ± 0.49 µg QE/mg extract. The flavonol content of the extracts was determined using the aluminum trichloride colorimetric method (AlCl_3_) at 440 nm. As shown in Table 1, the results indicate that the BUE exhibited higher flavonols (47.30 ± 0.51), followed by EAE (27.86 ± 1.45 µg QE/mg of extract).

The TPC of plant extracts depend on the type of extract, i.e., the polarity of the solvent used in extraction. The high solubility of phenols in polar solvents provides a high concentration of these compounds in the extracts obtained using polar solvents. The concentration of flavonoids in plant extracts depends on the polarity of the solvents used in the extract preparation [31]. Therefore, many studies have demonstrated that polar solvents give higher yields than non-polar solvents, since polar solvents have the ability to spread within the plant powder, reaching the vegetable matrix and therefore recovering the possible metabolites. In contrast, non-polar solvents, which are immiscible with water, do not have the ability to extract the maximum number of bioactive molecules because of the water contained in the plant tissue.

The anti-radical activity of the extracts toward the radical DPPH• was evaluated with spectrophotometry at 517 nm following the reduction of this radical, which is accompanied by a change from violet to yellow color. In this test the results were compared to the reference standards (BHA and BHT). The BUE showed the best anti-radical activity compared with the other extracts (IC_50_ = 59.38 ± 0.72 µg/mL); this activity was twice as low as BHT (IC_50_ = 22.32 ± 1.19 µg/mL) and eleven times as low as BHA (IC_50_ = 5.73 ± 0.41 µg/mL).

The scavenging activity towards the galvinoxyl radical was evaluated with spectrophotometry at 428 nm following the anti-radical reaction, which changes the solution’s color from dark yellow to light yellow. In this test the results were compared to the reference standards (BHA and BHT). From the values of IC_50_ (μg/mL) calculated from the inhibition percentage curves, it is noted that the BUE showed the highest scavenging activity with a value of (IC_50_ = 36.25 ± 0.72 μg/mL) followed by the EAE (IC_50_ = 97.72 ± 3.07μg/mL), CE (IC_50_ = 88.87 ± 1.86 μg/mL) and the CHE (IC_50_ > 200 μg/mL). The BUE’s activity was ten times lower than against BHT (IC_50_ = 3.32 ± 0.18 µg/mL) and six times lower than against BHA (IC_50_ = 5.38 ± 0.06 µg/mL). This test confirms the results of the previous test.

The antioxidant activity assessed using CUPRAC is based on the measurement of the absorbance at 450 nm, which indicates the reduction in the presence of an antioxidant of the stable complex neocuproine-copper (II) (blue color) to the stable complex neocuproine-copper (I) (orange color). Based on the results in Table 3, the BUE has the highest inhibitory activity of the samples studied, with a value of A_0.5_ = 71.80 ± 1.22 µg/mL, followed by the EAE, CE and CHE with values of A_0.5_ equal to 92.00 ± 4.85, 128.44 ± 4.14 and 188.33 ± 4.62 µg/mL, respectively (Tukey’s HSD test: *p*-value < 0.01). The BUE’s activity is seven times lower than that of BHT (A_0.5_ = 9.62 ± 0.87µg/mL) and twenty times lower than that of BHA (A_0.5_ = 3.64 ± 0.19 µg/mL) (Tukey’s HSD test: *p*-value < 0.01). The results of this test confirm the results of the first two tests. 

Potassium ferricyanide is reduced in the presence of an antioxidant to form potassium ferrocyanide, which then reacts with ferric chloride to form a blue-green ferrous iron complex, which has a maximum absorbance at 700 nm. The results are shown in Table 3. The results of the reducing potency test confirm the results of the first three tests: the BUE, with a value of A_0.5_ = 119.17 ± 0.29 µg/mL, showed the best activity compared to other extracts from the same plant (Tukey’s HSD test: *p*-value < 0.01). This activity is much lower than that of the two standards, ascorbic acid (A_0.5_ = 6.77 ± 1.15 µg/mL) and tannic acid (A_0.5_ = 5.39 ± 0.91 µg/mL), but is three times lower than the standard α-tocopherol (A_0.5_ = 34.93 ± 2.38 µg/mL). This result may be due to the presence of electron donor compounds, since the reducing-power method follows the mode of action of electron transfer (ET).

In the presence of an antioxidant, ferric iron (Fe^+3^) is reduced to ferrous iron (Fe^+2^), and the latter forms a stable complex with orange-red phenanthroline, which has a maximum absorbance at 510 nm. Compared to the other extracts, the BUE showed the best iron-reduction activity, with A_0.5_ = 20.29 ± 1.16 µg/mL, twenty times lower than BHA (A_0.5_ = 0.93 ± 0.07 µg/mL) and nine times lower than BHT (A_0.5_ = 2.24 ± 0.17 µg/mL). The results of this activity are consistent with the previous five methods.

ABTS (2,2′-azino-bis (3-ethylbenzothiazoline-6-sulphonic acid)) is a stable organic compound used in the evaluation of anti-radical activity, with a maximum absorbance at 734 nm. In the presence of an antioxidant donor of hydrogen, the cation ABTS•+ undergoes a reduction from blue-green to a neutral colorless state. The results of the ABTS test revealed that the BUE has the best anti-radical activity (IC_50_ = 49.52 ± 1.54 µg/mL) compared to other extracts from the same plant. Its activity is moderate compared to those of the two standards, BHT and BHA (IC_50_ = 1.29 ± 0.30, 1.81 ± 0.10 µg/mL, respectively). The results of the sixth test confirm the results of the previous tests.

The superoxide-radical-scavenging activity of the extracts was measured. As shown in Table 3, all the extracts have a scavenging activity against the superoxide radical and the highest activity was recorded for the BUE (IC_50_ = 13.61 ± 0.384 µg/mL), followed by the EAE (IC_50_ = 14.36 ± 0.90 µg/mL), the CE (IC_50_ = 21.91 ± 0.70 µg/mL) and finally the CHE (IC_50_ = 64.77± 2.37µg/mL) (Tukey’s HSD test: *p*-value < 0.01). Taking into account the high inhibition values of the extracts at the different concentrations, these results are almost within the range of the standards used, tannic acid and α-tocopherol (IC_50_ = 3.125 µg/mL).

Different plant extracts are potential sources of the natural chemical components responsible for antioxidant activities. It appears that the butanolic extract of *C. parviflora* shows a perfect antioxidant activity against DPPH, galvinoxyl, ABTS and superoxide free radicals as well as a reducing power in the CUPRAC, Fe^+2^-phenanthroline, and ferric-reducing tests. It should be noted that the order of efficacy of the studied extracts was similar in all the methods used, along with their polyphenol content, confirming that the effect of the extracts is completely consistent with the richness of these extracts in TPC.

Several studies showed that chronic diseases such as cancer, ageing and cardiovascular, inflammatory and neurodegenerative pathologies are associated with oxidative stress, a metabolic condition that causes cell degeneration. Antioxidant compounds present in fruits and vegetables appear to play a major role in the protection against oxidative stress. Besides fruits and vegetables, plant beverages such as coffee contribute to the dietary intake of antioxidants [32]. Epidemiological studies have shown relationships between the consumption of polyphenol-rich foods and the prevention of diseases such as cancer, coronary heart disease and osteoporosis, and the results of those studies have promoted an interest in polyphenols. Dietary polyphenols are thought to be beneficial for human health by exerting various biological effects such as free-radical scavenging, metal chelation, modulation of enzymatic activity, and alteration of signal transduction pathways [33]. The role of phenolic compounds has been widely shown in the protection against certain diseases due to their possible interaction with many enzymes and their antioxidant properties. Specifically, flavonoids are attributed various properties: antitumor, anti-radical, anti-inflammatory, analgesic, anti-allergic, antispasmodic, antibacterial, hepatoprotective, estrogenic and/or anti-estrogenic [34]. Polyphenols are associated with many physiological processes in the quality of food that occur when the plant is subjected to mechanical injuries. The ability of a plant species to resist attack by insects and microorganisms is often correlated with the content of phenolic compounds [35]. Due to the conjugated ring structure and the presence of hydroxyl groups, many phenolic compounds have the potential to act as antioxidants by hydrogenation or complexation with oxidizing species to scavenge or stabilize free radicals involved in the oxidation process [36]. Chlorogenic acid (CGA) is an important polyphenolic compound that occurs naturally in various agricultural products such as coffee, beans, potatoes, and apples [37]. It has been suggested that this compound exhibits an antioxidant property, and it has been reported that CGA inhibits vitamin A oxidation and protects against the oxidation of epinephrine in vitro. Recently, results from in vivo studies have suggested that CGA provides beneficial effects during ischemia-reperfusion injury of rat liver and paraquat-induced oxidative stress in rats [38]. Chlorogenic acids are phenolic compounds formed by the esterification of cinnamic acids. They exhibit various pharmacological properties. Its antioxidant, anti-obesity, anti-viral, anti-hypertension, antipyretic and anti-inflammatory properties are being studied by various researchers [39]. Chlorogenic acid is a biologically active polyphenol that is soluble in ethanol and acetone. Chlorogenic acids are present in abundance in green coffee beans. They are also found in potatoes, prunes and bamboo. Its wide availability makes it economical and aids its easy application. In addition, it has been found that chlorogenic acids have the capability to modulate lipid metabolism and glucose in both genetic and lifestyle-related metabolic disorders [40]. Chlorogenic acid and caffeic acid have vicinal hydroxyl groups on an aromatic residue, and they exhibit antimutagenic, anti-carcinogenic and antioxidant activities in vitro, which is attributed to the scavenging of reactive oxygen species (ROS) [41]. Chlorogenic acid is a principle phenolic compound in nectarine fruit pulp and has strong antioxidant activity, which is positively correlated with ROS-scavenging ability in peach and nectarine fruit. However, little is known about the effects of polyphenols on fruit proteins. In previous studies, researchers have demonstrated that exogenous CHA can significantly delay the senescence of nectarine fruit [42].

In total, ten flavonoids representing four major categories were screened to evaluate their antioxidant activity. Rutin showed the highest level of free-radical-scavenging capacity, followed by kaempferol, luteolin, quercetin, apigenin, hesperidin, sinensetin, naringenin, naringin and 3,5,6,7,8,3’,4’-heptamethoxyflavone [43]. However, quercetin and quercetin 3-*O*-glucoside displayed antioxidant activities that were consistent with the reported activities of these flavonoids in the literature [44]. The results obtained by Razavi [45] showed that quercetin 3-*O*-glucoside exhibits a high antioxidant effect with an RC50 of 22 µg/mL, has low cytotoxicity and has no antibacterial effects. Quercetin 3-*O*-glucoside also exhibits a high phytotoxic effect, with an IC50 value of 282.7 µg/mL.

#### Antimicrobial Activity

**Figure 14:**
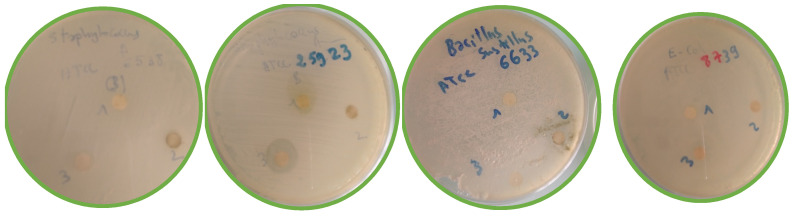

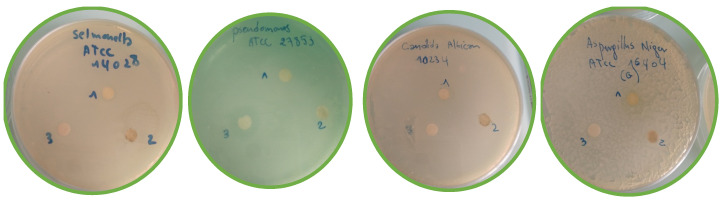
Photographs of the antibacterial activity results according to the agar disk-diffusion method of *C. parviflora*. 1. Standard (solvent), 2. 20 mg/mL, 3. 30 mg/mL.

The antimicrobial activities of the *C. parviflora* extract were evaluated using the well-diffusion technique, and the results are shown in Table 4 and Figure 14.

The antimicrobial activities of the methanolic extract against bacterial reference and pathogenic fungi were assessed (Table 4). According to the obtained data, the disc method made it possible to determine the action of the plant extracts dissolved in DMSO on the different strains. These tests revealed a good antimicrobial effect of the methanolic extract against most of the tested microbes expect *Escherichia coli* and *Candida albicans*. Broadly speaking, we found values for the inhibition zone diameter (IZD, mm) ranging from 7.00 to 12.12 mm and 8.03 to 15.23 mm, respectively, for the 20 and 30 mg/mL extract concentrations. The extracts at a concentration of 30 mg/mL are active and exhibit antimicrobial activity by inhibiting the in vitro growth of microbial germs: *S. aureus* ATCC6538 (IZD, 8.64 and 7.00 mm), *S. aureus* ATCC25923 (IZD, 13.22 and 12.00 mm), *Salmonella* (IZD, 8.03 and 7.00 mm), *A. niger* (IZD, 8.22 and 7.00 mm) and *Pseudomonas* sp. (IZD, 15.23 and 12.12 mm). The marked observation to emerge from the data comparison was the potential inhibition effect of *C. parviflora* extract against *Staphylococcus aureus,* a leading cause of skin and soft tissue infections, and *Pseudomonas* sp., which particularly causes infections in the blood, lungs (pneumonia) or other parts of the body after surgery. This is in good agreement with the findings of Naeim et al. [46], who reported a high antibacterial activity of the methanol extract from *C. pumilio* roots and aerial parts against *Staphylococcus aureus* and *Acinetobacter baumannii* strains (MIC, 62.50 μg/mL and 250 μg/mL, respectively). Furthermore, Karamenderes et al. [47] demonstrated high antiprotozoal (*Plasmodium falciparum* and *Leishmania donovani*) and antimicrobial (*Candida albicans, Candida glabrata, Candida krusei, Cryptococcus neoformans, Mycobacterium intracellulare, Aspergillus fumigatus*, and methicillin-resistant *Staphylococcus aureus*) activities of ten *Centaurea* L. species growing in Turkey. The data obtained are broadly consistent with the major trends reported in the literature [48]. To cure diseases in humans and animals brought on by microorganisms that are medication-resistant, new antimicrobial medicines are required. Additionally, there is a persistent desire from consumers for “natural” and/or “preservative-free” foods and cosmetics that are microbiologically safe [49]. Plants are a significant source of bioactive constituents including phenols, aromatic components, terpenoids, sterols, essential oils, alkaloids, tannins and anthocyanins that play a significant role in the treatment of many diseases [50]. Methanolic extracts can potentially be used as natural antimicrobial and antioxidant agents against infectious diseases in humans and for the preservation of food products. The development of such natural agents will also help to solve environmental problems caused by synthetic products and synthetic drugs such as pollution and resistance of certain microorganisms.

Phenolic acids have recently gained substantial attention due to their various practical, biological and pharmacological effects. The extended list of chlorogenic acids contains approximately 400 compounds. Chlorogenic acid is an important and biologically active dietary polyphenol, playing several important and therapeutic roles such as antioxidant activity, antibacterial, hepatoprotective, antiviral and antimicrobial [51].

## 3. Material and Methods

### 3.1. Plant Materials

The aerial parts of *C. parviflora* Desf. were harvested in Djebel Djedoug (forest of Dréat, Msila, northern Algeria in June 2021 (altitude 1179 m, latitude 35.8838° or 35°53′2″ North, longitude: 4.3284° or 4°19′42″ East). The taxonomic identification of plant materials was confirmed by Dr. M.D. Miara at the Department of Biology, University of Tiaret, using *Flora of Algeria* [13], and an herbarium specimen was archived in the Herbarium of Tiaret University, Tiaret, Algeria.

### 3.2. Extraction and Fractionation

#### Chemicals

Chloroform, ethyl acetate, butanol, Folin–Ciocalteu reagent, sodium bicarbonate, aluminum chloride, tannic acid, ascorbic acid, 2,2-diphenyl-1-picrylhydrazyl (DPPH), 2,2′-azino-bis(3-ethylbenzothiazoline-6-sulfonic acid) diammonium salt (ABTS), potassium persulfate, hydrogen peroxide, disodium hydrogen phosphate, sodium phosphate monobasic dihydrate, ferrous chloride, trichloroacetic acid (TCA), nitroblue tetrazolium (NBT), ferric chloride, butylated hydroxyanisol (BHA) and butylated hydroxytoluene (BHT) were supplied by Sigma-Aldrich (St. Louis, MO, USA).

The extraction of the plant powder was conducted according to Zaak et al. [52] and Mammeri et al. [53]. The first step consisted of a solid–liquid extraction, where an amount of 50 g of aerial-art powder was macerated at room temperature three times with 300 mL methanol–water (80%). The combined filtrates were evaporated under low pressure with a rotary evaporator (40 °C) to yield the crude methanolic extract (CE). Then, the crude extract was subjected to liquid–liquid extraction (fractionation) using organic solvents of increasing polarity: chloroform giving chloroform extract (CHE), ethyl acetate giving ethyl acetate extract (EAE) and *n*-butanol giving butanol extract (BUE). All solvents were removed using a rotary evaporator.

### 3.3. Chromatographic Analysis

#### 3.3.1. Qualitative Analysis with Thin-Layer Chromatography (TLC)

Thin-layer chromatography is a rapid analytical technique and method for separating compounds. It applies to pure molecules and extracts. It is a rapid physicochemical method based on the phenomena of adsorption, interaction and polarity. Compounds can be characterized according to their Rf (retention factor). It allows us to obtain a general idea of the metabolites present and allows an easy and fast control of the purity of a compound when the operating conditions are well-determined. The methanolic extract was checked with TLC on analytical plates over silica gel. Aluminum plates coated with Merck 60F254 silica gel were used. Solvent systems of different polarities were prepared (Table 5).

The methanolic extract was applied to pre-coated TLC plates using capillary tubes and developed in a TLC chamber using mobile phases. The developed TLC plates were air-dried and observed under visible light and ultra violet light (UV, 254 and 366 nm). The movement of the analyte was expressed as its retention factor (Rf):

Rf = Distance travel by solute/Distance travel by solvent (Rf—Retention factor)

#### 3.3.2. HPLC Analysis

The phenolic constituents in the butanolic fraction of *C. parviflora* were investigated using LC-MS analysis and tentatively identified by comparison with the literature. The HPLC analysis was performed using the Agilent 6420 series triple-quadrupole double-MS instrument with the latest generation of high-performance liquid chromatography, the HPLC 1260 infinity LC, equipped with a vacuum degreaser, Infinity 1260 automatic injector, double-piston pump in series and ultra-sensitive diode (DAD) baratte UV detector. The column used for chromatographic separation was a Zorbax Eclipse Plus C18 (1.8 μm, 150 mm × 4.6 mm) (Agilent Technologies, Palo Alto, CA, USA). In separating the compounds from the butanolic fraction of *C. parviflora*, the rate of flux used was 0.80 mL/min and the analysis was performed at room temperature. A gradient elution was run, utilizing as eluent A water with 0.1% formic acid and as eluent B acetonitrile. The following multistage linear gradient was applied: 0 min, 15% B; 35 min, 95% B; 40 min, 95% B; 55 min, 15% B and, finally, a conditioning cycle of 5 min, with the same conditions for the following analysis. The injection volume was 10 µL. The flow rate of the mobile phase was 0.4 mL/min, and the temperature of the column was maintained at 40 °C. The mass spectrometer was operated in the negative ion mode with a capillary voltage of 4000 V and the drying gas stream was the nitrogen.

### 3.4. Antioxidant Activities

#### 3.4.1. Total Phenolic Content (TPC)

The TPC of extracts was estimated with the method of Müller [54]. A mixture of 200 µL sample and 1 mL of 10% Folin–Ciocalteu reagent was incubated for 4 min, and then 800 µL of sodium carbonate (7.5%) was added. All reactants were finally incubated for 2 h and the absorbance was taken at 765 nm against the corresponding blank. Gallic acid was used as a standard and a calibration curve was prepared in the same conditions (y = 0.0034 x + 0.1044; *R*^2^ = 0.9972). The results were expressed as µg GAE/mg dry extract.

#### 3.4.2. Total Flavonoid Content (TFC)

The total flavonoid content was evaluated following the aluminum trichloride method of Topçu [55] with some modifications. An amount of 300 µL of extract was mixed with the same volume of aluminum trichloride (2%) and incubated for 15 min. The absorbance was measured at 430 nm. For this assay, quercetin was used to prepare a calibration curve in the same conditions (Y = 0.0071x + 0.0274; *R*^2^ = 0.9985). The results were expressed as QE/mg.

#### 3.4.3. Total Flavonol (TFOL) Content

To evaluate the flavonol content of our extracts, 1 mL of sample and 1 mL of aluminum trichloride (2%) were mixed with 1.5 mL of aqueous sodium acetate (5%) and incubated at 25 °C for 2.5 h. The absorbance was then read at 440 nm and the results were expressed as RE/mg dry extract using a calibration curve prepared with rutin (y = 0.0122x + 0.0179; *R*^2^ = 0.9991) [56].

#### 3.4.4. DPPH Free-Radical Scavenging

The DPPH free-radical-scavenging activity was assessed using the Blois method [57]. Briefly, in a 96-well microplate (PerkinElmer Multimode Plate Reader EnSpire, Waltham, MA, USA), 40 µL of the sample at different concentrations was added to 160 µL of freshly prepared DPPH (0.1 mM). The absorbance of the reaction mixture was measured using spectrophotometry at a wavelength of 517 nm after an incubation of 30 min at room temperature in darkness. The percentage of inhibition was calculated using the following equation:

% Inhibition = (Ac − At/Ac) × 100

where Ac is the absorbance of the control and At is the absorbance of the test.

The inhibitions obtained were plotted against the sample concentrations and the resulting plots were used to calculate the IC_50_ (the concentration of the sample that reduced 50% of the DPPH).

#### 3.4.5. Galvinoxyl Free-Radical Scavenging

The galvinoxyl free-radical-scavenging activity was determined using Shi’s [58] method. In a 96-well microplate, a volume of 160 µL of galvinoxyl (0.1 mM) was added to 40 µL of sample prepared at different concentrations. After 120 min, the absorbance was measured at a wavelength of 428 nm. The inhibition percentage was calculated according to the following equation:

% Inhibition = (Ac − At/Ac) × 100

where Ac is the absorbance of the control and At is the absorbance of the test.

The inhibitions obtained were plotted against the sample concentrations and the resulting plots were used to calculate the IC_50_ (the concentration of the sample that reduced 50% of the calvinoxyl).

#### 3.4.6. ABTS-Scavenging Assay

The radical-scavenging activity was evaluated using the stable cation radical ABTS, as described by Re et al. [59]. The ABTS radical was generated by mixing the ABTS solution (7 mM) with 13.24 mg of potassium persulfate for 16 h. The resulting solution was refrigerated, then diluted to reach an absorbance of 0.7 ± 0.02 at 734 nm. A 100 µL volume of the sample was mixed with 1.9 mL of ABTS^•+^ solution and incubated. After 7 min, the absorbance was measured at 734 nm. BHA and BHT were used as standard compounds. The ABTS inhibition was calculated using the following formula:

% Inhibition = (Ac − At/Ac) × 100

where Ac is the absorbance of the control and At is the absorbance of the test.

The inhibitions obtained were plotted against the sample concentrations and the resulting plots were used to calculate the IC_50_ (the concentration of the sample that reduced 50% of the ABTS).

#### 3.4.7. Superoxide-Radical-Scavenging Activity (O_2_^•−^)

The activity was determined according to the method of Elizabeth and Rao [60]. In a 96-well plate with a volume of 200 μL for each well, 40 μL of the sample solution at different concentrations (3.125, 6.25, 12.5, 25, 50, 100 and 200 μg/mL), 130 μL of alkaline DMSO (20 mg of NaOH in 100 mL DMSO), and 30 μL of NBT (10 mgNBT in 10 mL of distilled water). Absorbances are measured instantaneously at room temperature at 560 nm (results were expressed as the average of three separate measurements ± standard deviation).

A blank was prepared for each concentration. Tannic acid and ascorbic acid were used as reference compounds. Percent inhibition of superoxide was calculated according to the following equation:

% Inhibition = (Ac − At/Ac) × 100

where Ac is the absorbance of the control and At is the absorbance of the test.

The inhibitions obtained were plotted against the sample concentrations and the resulting plots were used to calculate the IC_50_ (the concentration of the sample that reduced 50% of the superoxide).

#### 3.4.8. CUPric Reducing Antioxidant Capacity (CUPRAC)

This activity was determined with the method of Apak et al. [61]. In a 96-well plate, 40 μL of the sample was added to 60 μL of ammonium acetate buffer (1 M, PH = 7), 50 μL of neocuproine (7.5 mM) and 50 μL of CuCl_2_ (10 mM). After one hour, the absorbance was recorded at 450 nm with a microplate reader. The antioxidant activity results were calculated as A_0.5_ (µg/mL).

#### 3.4.9. Ferric-Reducing Power

The reducing power of the extracts was determined according to the methods of Bouratoua [62]. A volume of 10 μL of the extract at different concentrations was mixed with 40 μL of phosphate buffer solution (0.2 M, pH = 6.6) and 50 μL of a potassium ferricyanide [K_3_Fe (CN)6] solution (1%). The mixture was incubated at 50 °C for 20 min. Then, 50 µL of trichloroacetic acid (10%) was added to stop the reaction and the whole was centrifuged at 3000 r/min for 10 min. Finally, 50 µL of the supernatant solution was mixed with 50 µL of distilled water and 10 µL of FeCl_3_ (0.1%) and the absorbance was recorded at 700 nm after incubation for 10 min. An increase in the absorbance corresponds to an increase in the reducing power of the test extracts.

#### 3.4.10. Phenanthroline Test

The reduction activity in terms of the formation of the Fe^+2^-phenanthroline complex of the extracts was measured using the method described by Szydlowska-Czerniaka [63] and Aissani et al [64]. A volume of 10 μL of various concentrations of extract or standards was added to the reaction mixture containing deoxyribose, then 50 μL FeCl_3_ (0.2%), 30 µL phenanthroline (0.5%) and 110 µL of the methanol were added. The mixture was vigorously agitated and incubated for 20 min in the oven at 30 °C. The absorbance was determined at 510 nm. The results were calculated as A_0.5_ (μg/mL), corresponding to the concentration indicating 0.50 absorbance.

#### 3.4.11. Microorganisms

A total of 8 microbial cultures belonging to bacteria, yeast and fungi species were used in this study. The antimicrobial activity of the methanolic extract of *C. parviflora* was evaluated In vitro with the growth of several bacterial, fungal and yeast strains, namely *P. aeruginosa*, *S. aureus*, *B. subtilis*, *E. coli*, *Salmonella*, *Aspergillus niger* and *Candida albicans*. The microorganisms were provided by the laboratory of the Department of Microbiology, M’sila University, M’sila, Algeria.

The crude methanolic extract was diluted to a final concentration of 30 mg/mL, then sterilized by filtration (0.45 m) with Millipore filters. The antimicrobial tests were carried out using the disc-diffusion method, using 100 µL of suspension containing 10^8^ CFU/mL of bacteria or 10^6^ CFU/mL of yeast spread on nutrient agar (NA) and Sabouraud dextrose agar (SDA), respectively. The discs (6 mm in diameter from Whatman N° 1 filter paper) were impregnated with 10 µL of the methanolic extracts placed on the surface of the agar. Negative controls were prepared using the same solvents. The Petri plates were placed at a low temperature (+4 °C) for 15 to 30 min to allow the extracts to diffuse into the agar before the bacteria began to multiply. After 24 h of incubation at 37 °C for bacterial strains and 48 h for yeast and fungi isolates, the diameter of inhibition (mm) surrounding the disc was measured using a graduated ruler. The diameter is proportional to the sensitivity of the germ studied. Each assay in this experiment was repeated twice.

### 3.5. Statistical Analyses

The results of the tests carried out are expressed as an average ± SD of analyses in three tests. The values of IC_50_ (50% inhibition concentration) and A_0.5_ (the concentration indicating 0.50 absorbance) are calculated using the linear regression method from the two curves: [% inhibition = f (concentration)] for IC_50_ and [Absorbance = f (concentration)] for A_0.5_. The data were tested first for normality (Kolmogorov–Smirnov test) and equality of variance (Bartlett test) to fulfill the requirements of parametric analyses, and log-transformations were applied when these assumptions were not met. One-way analysis of variance (ANOVA) and Tukey’s honestly significant difference (HSD) test were performed with the software STATISTICA v.8 in order to test for the significant global and pairwise comparisons, respectively.

## 4. Conclusions

The extraction of the aerial parts of *C. parviflora* using various solvents led to four extracts who’s phenolic, flavonoid and flavonol contents differ. The methanolic extract had a good antimicrobial effect, making it a product with potential interest in the pharmaceutical industries. Moreover, the antioxidant activity of the different extracts was studied in vitro using seven techniques. The best antioxidant capacity was observed with the BUE and EAE. The results showed that there is a divergent correlation between the TPC of the extracts and its activity as an antioxidant and antimicrobial. This relationship became clearer after the phytochemical analysis, which showed the existence of compounds with specialized effects.

The TLC of the CE confirmed the presence of bioactive compound such as flavonoids, polyphenols, etc. In addition, the LC-MS analysis of the BUE of *C. parviflora* allowed us to identify eight compounds including six phenolic acids and two flavonoids: quinic acid, five chlorogenic acid derivatives, rutin and quercetin-3-*O*-glucoside. This preliminary investigation reveals that the extracts of *C. parviflora* have a good biopharmaceutical activity. The BUE possesses an interesting potential for pharmaceutical/nutraceutical applications.

## Figures and Tables

**Table 1:** Phytochemical analysis of methanolic extract of *C. parviflora* with thin-layer chromatography.

N	Chemical Name	Solvent System	Rf Value	Spot Color	References
1	Flavonoids	Toluene/	0.12	Pale pink	[26]
		Chloroform/	0.18	Light blue	
		Methanol (4/4/1) (*v*/*v*/*v*)	0.56	Pink	
			0.6	Pink	
			0.65	Blue	
			0.78	Purple	
			0.95	Light blue	
2	Flavonoids	Chloroform/Methanol	0.29	Mauve	[27]
		(10/1) (*v*/*v*)	0.73	Pale pink	
3	Polyphenols/Flavonoids	Ethyl Acetate/Methanol	0.12	yellowish blue	[28]
		/Water (8/1/1) (*v*/*v*/*v*)	0.24	Grey	
			0.34	Grey	
			0.39	Grey	
			0.6	Yellow	
			0.44	Fluorescent White Blue Gray	
			0.74		
4	Polyphenols	Methanol/Water (7/3) (*v*/*v*)	0.88	dark gray	[29]
5	Flavonoids	Butanol/Acetic acid/Water (40/10/50) (*v*/*v*/*v*)	0.31	Grey	-
			0.4	dark gray	
			0.52	Fluorescent white blue	

**Table 2:** Compounds of the *C. parviflora* butanol extract identified with LC-MS.

Pic N	Compounds	Retention Time (min)	Molecular Formula	Experimental *m*/*z*	Calculated *m*/*z*	Ionization Mode
1	Quinic Acid	0.68	C_7_H_12_O_6_	191.00	191.05	Neg
2	Chlorogenic acid derivative	0.72	C_16_H_18_O_9_	353.00	353.08	Neg
3	Chlorogenic acid derivative	1.03	C_16_H_18_O_9_	353.00	353.08	Neg
4	Chlorogenic acid derivative	1.20	C_16_H_18_O_9_	353.00	353.08	Neg
5	Chlorogenic acid derivative	1.60	C_16_H_18_O_9_	353.00	353.08	Neg
6	Rutin	3.60	C_27_H_30_O_16_	609.00	609.14	Neg
7	Quercetin 3-*O*-glucoside	4.26	C_21_H_20_O_12_	463.00	463.08	Neg
8	Chlorogenic acid derivative	5.73	C_30_H_10_O_14_	593.00	593.00	Neg

**Table 3:** In vitro antioxidant activity using different assays of *C. parviflora* extracts (*n* = 3, *log*-transformed data, Tukey’s HSD test: *p*-value < 0.01 (a,b and b,c); *p*-value < 0.0001 (a,c)).

	DPPH (IC_50_ µg/mL)	Galvinoxyl (IC_50_ μg/mL)	ABTS (IC_50_ μg/mL)	CUPRAC (A_0.5_, μg/mL)	RP Phenanthroline (A_0.5_, μg/mL)	RP (A_0.5_, μg/mL)	Superoxide (IC_50_ μg/mL)
CE	160.92 ± 5.11 ^(a)^	88.87 ± 1.86 ^(a)^	162.39 ± 0.15 ^(a)^	128.44 ± 4.14 ^(a)^	46.56 ± 1.54 ^(a)^	˃200	21.91 ± 0.70 ^(a)^
CHE	˃200	˃200	˃200	188.33 ± 4.62 ^(b)^	75.67 ± 0.29 ^(a)^	˃200	64.77 ± 2.37 ^(b)^
EAE	144.75 ± 1.17 ^(a)^	97.72 ± 3.07 ^(a)^	124.22 ± 0.61 ^(a)^	92.00 ± 4.85 ^(a)^	56.56 ± 3.34 ^(a)^	193.67 ± 1.44 ^(a)^	14.36 ± 0.90 ^(a)^
BUE	59.38 ± 0.72 ^(b)^	36.25 ± 0.42 ^(b)^	49.52 ± 1.54 ^(b)^	71.80 ± 1.22 ^(a)^	20.29 ± 1.16 ^(b)^	119.17 ± 0.29 ^(b)^	13.61 ± 0.38 ^(a)^
BHT	22.32 ± 1.19 ^(b)^	3.32 ± 0.18 ^(c)^	1.29 ± 0.30 ^(c)^	9.62 ± 0.87 ^(c)^	2.24 ± 0.17 ^(c)^	nd	nd
BHA	5.73 ± 0.41 ^(c)^	5.38 ± 0.06 ^(c)^	1.81 ± 0.10 ^(c)^	3.64 ± 0.19 ^(c)^	0.93 ± 0.07 ^(c)^	nd	nd
Tannic acid	nd	nd	nd	nd	nd	5.39 ± 0.91 ^(c)^	˂3.125
Ascorbic acid	nd	nd	nd	nd	nd	6.77 ± 1.15	˂3.125

BHT: Butylhydroxyltoluene, BHA: Butylhydroxyanisole, nd: not detected. n.d. not determined.

**Table 4:** Antimicrobial and antifungal activities of *C. parviflora* methanolic extract.

Microorganisms Tested		Extract Concentration	
		20 mg/mL	30 mg/mL
		Inhibition Zone Diameter (IZD, mm)	
*Staphylococcus aureus* ATCC6538	Gram+	7.00	8.64
*Staphylococcus aureus* ATCC25923	Gram+	12.00	13.22
*Bacillus subtilis* ATCC6633	Gram+	NA	NA
*Escherichia coli* ATCC8739	Gram−	NA	NA
*Salmonella sp.* ATCC14028	Gram−	7.00	8.03
*Pseudomonas* sp. ATCC27853	Gram−	12.12	15.23
*Candida albicans* ATCC10234	Fungus	NA	NA
*Aspergillus niger* ATCC16404	Fungus	7.00	8.22

NA: no activity.

**Table 5:** Yield and contents of phenolic, flavonoid and flavonol compounds of *C. parviflora* extracts. Different letters indicate significant differences (*n* = 3, *log*-transformed data, Tukey’s HSD test: *p*-value < 0.01 (a,b and b,c); *p*-value < 0.001 (a,c)).

Extracts	Extraction Yield (%)	TPC (µg GAE/mgE) *	TFC (µg QE/mg E) *	TFOL (µg RE/mg E) *
Crude extract (CE)	2.36	113.51 ± 2.95 ^(a)^	24.49 ± 0.49 ^(a)^	20.03 ± 0.63 ^(a)^
Chloroform extract (CHE)	1.45	105.47 ± 1.35 ^(a)^	17.78 ± 0.21 ^(a)^	22.89 ± 1.33 ^(a)^
Ethyl Acetate Extract (EAE)	0.95	136.94 ± 2.94 ^(b)^	20.83 ± 0.21 ^(a)^	27.86 ± 1.45 ^(a)^
Butanol Extract (BUE)	0.83	175.27 ± 2.79 ^(c)^	59.89 ± 0.91 ^(b)^	47.30 ± 0.51 ^(b)^

* Values are expressed as means ± SD of three parallel measurements, GAE: Gallic acid equivalent, QE: Quercetin equivalent, RE: Rutin equivalent, TPC: Total phenolic content, TFC: Total flavonoid content, TFOL: Total flavonol.

## Data Availability

All the data are included in the paper.

## Sample Availability

Samples of all the compounds are available from the authors.
